# Barriers to Mental Health Treatment in the Saudi National Mental Health Survey

**DOI:** 10.3390/ijerph17113877

**Published:** 2020-05-30

**Authors:** Abdulaziz S. Alangari, Sarah S. Knox, Alfgeir L. Kristjansson, Sijin Wen, Kim E. Innes, Lisa Bilal, Abdulhameed Alhabeeb, Abdullah S. Al-Subaie, Yasmin A. Altwaijri

**Affiliations:** 1 Department of Epidemiology, School of Public Health, West Virginia University, Morgantown, WV 26506, USA; sknox@hsc.wvu.edu (S.S.K.); kinnes@hsc.wvu.edu (K.E.I.); 2 Department of Epidemiology and Biostatistics, College of Public Health and Health Informatics, King Saud bin Abdulaziz University for Health Sciences, Riyadh 11481, Saudi Arabia; 3 Department of Social and Behavioral Sciences, School of Public Health, West Virginia University, Morgantown, WV 26506, USA; alkristjansson@hsc.wvu.edu; 4 Department of Biostatistics, School of Public Health, West Virginia University, Morgantown, WV 26506, USA; siwen@hsc.wvu.edu; 5 Biostatistics, Epidemiology and Scientific Computing Department, King Faisal Specialist Hospital and Research Centre, Riyadh 11211, Saudi Arabia; lbilal@kfshrc.edu.sa (L.B.); yasmint@kfshrc.edu.sa (Y.A.A.); 6 King Salman Center for Disability Research, Riyadh 12512, Saudi Arabia; 7 SABIC Psychological Health Research & Applications Chair (SPHRAC), College of Medicine, King Saud University, Riyadh 12372, Saudi Arabia; prof.subaie@gmail.com; 8 National Center for Mental Health Promotion, Ministry of Health, Riyadh 11525, Saudi Arabia; aalhabeeb@ncmh.org.sa; 9 Edrak Medical Center, Riyadh 12281, Saudi Arabia

**Keywords:** barriers to treatment, mental disorders, dropout rates, unmet need for treatment, mental services, Saudi National Mental Health Survey (SNMHS), World Mental Health (WMH) survey initiative

## Abstract

*Objectives*: To examine barriers to initiation and continuation of treatment among individuals with common mental disorders in the Saudi National Mental Health Survey (SNMHS). *Methods*: The SNMHS is a community-based epidemiological survey in a nationally representative household sample of respondents aged 15–65 in the Kingdom of Saudi Arabia. The World Health Organization Composite International Diagnostic Interview (CIDI) 3.0 was used. Predictors of barriers to treatment were analyzed with multivariable logistic regression. *Results*: Among participants with a 12-month DSM-IV/CIDI disorder (*n* = 711), 86.1% reported no service use. Of those (*n* = 597), 50.7% did not think they needed any help (categorized as “low perceived need”) and 49.3% did perceive need. Of those who perceived need (*n* = 309), the majority (98.9%) reported attitudinal barriers to initiation. In contrast, 10.3% of those with a perceived need reported structural barriers. Respondents who were previously married or indicated below-average income were more likely to believe they needed help. *Conclusions*: Among people with a diagnosed mental disorder, low perceived need and attitudinal barriers are the primary barriers to mental health treatment in the KSA. The results suggest that addressing poor mental health literacy may be essential factor in reducing the unmet need for mental health treatment in the KSA.

## 1. Introduction

Mental disorders represent a growing disease burden globally, with more than 1 billion people currently suffering from mental health- or substance use disorders [1,2]. They account for over 32% of years lived with disability (YLDs), and an estimated 13% of disability-adjusted life-years (DALYs) worldwide [3]. Although health care expenditures continue to increase worldwide with an annual growth rate of 4.3% from 2000 to 2017 [4], a large proportion of individuals with mental disorders ultimately remain untreated [5,6,7]. Moreover, among those who do seek mental health services, many drop out before completing the recommended course of treatment [8]. 

The World Health Organization (WHO) World Mental Health (WMH) Survey Initiative has conducted epidemiological surveys of common mental disorders in more than 30 countries [9,10], including the Kingdom of Saudi Arabia (KSA). Findings from the WMH surveys have indicated an estimated overall prevalence of lifetime DSM-IV disorders to be 18.1–36.1% and 12-month prevalence to be 9.8–19.1% [11]. Of survey respondents who have been diagnosed with a mental health disorder, only 27.9% reported receiving mental health treatment, and of those who have sought help, 12.8% did not complete treatment as recommended [12]. Lack of perceived need for treatment is the most commonly cited reason for failure to both initiate and continue treatment, followed by attitudinal barriers (e.g., stigma, wanting to handle the problem on their own), and, less commonly, structural barriers (e.g., financial issues, access) [12]. 

Untreated mental disorders and early treatment termination not only increase the risk for chronic impairment, poor health-related quality of life, and reduced educational attainment, but are associated with serious economic and societal burdens [5,13,14] and ultimately a significant contributor to increased healthcare utilization and costs [15]. Identifying these barriers to treatment is critical for the development, design, and improvement of access to mental health services [16]. 

The Kingdom of Saudi Arabia (KSA) is classified as a high-income country [17]. Its total health expenditures comprise around 4.7% of the country’s gross domestic product (GDP) compared to the USA (17.1%), Australia (9.4%), UK (9.1%), and Singapore (4.9%) [18]. The Saudi Ministry of Health is the largest provider of healthcare services in the KSA, and they provide cost-free psychiatric services [19]. As participating investigators in the (WMH) surveys, the KSA researchers recently completed the Saudi National Mental Health Survey (SNMHS), a nationally representative study of the KSA population. Previous SNMHS assessments estimated that 20.2% of the population had at least one 12-month DSM-IV/CIDI disorder [20]. These estimates are suggestive of an unmet need for mental health services in the KSA [21], with only 13.7% of respondents diagnosed with a 12-month disorder reporting to having received treatment. 

Despite this, there is a lack of research investigating potential reasons for why Saudis that people with mental health disorders do not seek treatment. To help address this gap, the goal of this study was to examine the perceived need, as well as structural, and attitudinal barriers to service initiation, and to the continuation of mental health treatment in the Kingdom of Saudi Arabia using data from the SNMHS. 

## 2. Materials and Methods 

### 2.1. Sample

The SNMHS is a nationally-representative face-to-face household survey of respondents 15 to 65 years in the KSA that was conducted between 2011 and 2016. Those who were Saudi citizens and able to speak Arabic were included in the study. Excluded from the sampling frame were the two administrative areas Jazan and Najran, due to security concerns resulting from the political conflict along the Saudi borders at the time of the survey. Respondents were selected using a stratified, multistage probability sample that was proportionate to the 2010 estimated population by the General Authority for Statistics in the KSA.

**Figure 1:**
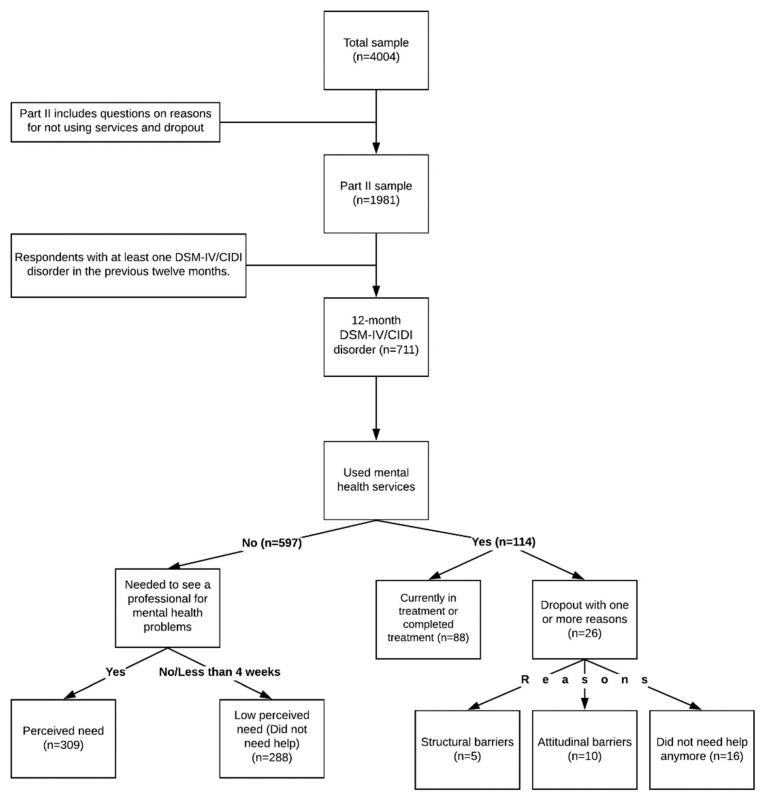
Study sample flow chart.

The interviews were divided into two parts. In Part I the core diagnoses were determined for all respondents (*n* = 4004), while Part II (*n* = 1981) assessed risk factors, correlates, service use, and other noncore disorders of participants who had reported any lifetime disorder in Part I, and a probability subsample of other Part I respondents (Figure 1). 

The estimated overall response rate for the SNMHS was 61% [20]. Informed written consent was obtained before the interview, and recruitment procedures were approved through the institutional review board of the Office of Research Affairs at King Faisal Specialist Hospital and Research Center (RAC#: 2091093). Details about other SNMHS survey methods (training procedures, translation protocols, and quality control procedures) for interviewers can be found elsewhere [22,23,24,25]. 

### 2.2. Measures

#### 2.2.1. Diagnostic Assessment of 12-Month Mental Disorders

The SNMHS survey uses the World Health Organization Composite International Diagnostic Interview (CIDI) version 3.0, a fully structured lay-administered diagnostic interview, to generate its diagnoses [9]. The CIDI is based on the American Psychiatric Association’s Diagnostic and Statistical Manual of Mental Disorders (DSM-IV) and the WHO International Classification of Disease (ICD 10) criteria [9]. DSM-IV organic exclusion was applied without diagnostic hierarchy rules for diagnoses. 

The 12-month (CIDI) disorders include: *mood disorders* (bipolar I and II disorders, subthreshold bipolar disorder, and major depressive disorder), *anxiety disorders* (panic disorder, agoraphobia without panic disorder, social phobia, generalized anxiety disorder, posttraumatic stress disorder, obsessive-compulsive disorder and adult separation anxiety disorder), *disruptive behavior disorders* (attention-deficit/hyperactivity disorder, conduct disorder, intermittent explosive disorder), *eating disorders* (anorexia nervosa, bulimia nervosa, binge-eating disorder), and *substance use disorders* (alcohol and drug abuse and dependence). 

Only those with at least one DSM-IV/CIDI disorder in the past twelve months were included in the analyses. An important point to mention is that the current DSM-V [26] changed the posttraumatic stress disorder (PTSD) from being an anxiety disorder to trauma- and stress-related disorders [27]. For the purposes of this paper, we are using the earlier classification of PTSD. 

#### 2.2.2. Levels of Disorder Severity

Criteria for disorder severity classifications were used similar to other WMH surveys [28]. Respondents with 12-month disorders were defined as *severe* if they were diagnosed with bipolar I, attempted suicide in concurrence with a core mental disorder, substance dependence (alcohol or drugs) that included physiological dependence symptoms, or had exhibited at least one core mental disorder with a high score of functional impairment in at least three of four areas in the modified version of the Sheehan Disability Scales (SDS) [29]. If respondents were not defined as severe, then they were classified as *moderate* if they had at least one mental disorder plus substance dependence without physiological dependence symptoms, or moderate impairment in any SDS domain. All other respondents were classified as *mild* if they had been diagnosed with any mental disorder in the past year.

#### 2.2.3. Use of Services

The 12-month service use was evaluated by asking respondents with 12-month disorders if they visited any professionals for problems with mental health, emotions, nerves, alcohol, or drug use. The list of providers included *psychiatrists*, *mental health professionals* (psychologist, other nonpsychiatric mental health professionals, social workers, or mental health counselors), *general medical providers* (primary care physicians or other general physicians), *human services professionals* (religious or spiritual advisors, or counselors in a setting different than a specialty mental health setting), and *complementary and alternative medicine (CAM)* practitioners (e.g., any other type of healers, such as herbalists, chiropractors, or were participants in self-help or support groups).

#### 2.2.4. Barriers for Not Using Services and Reasons for Treatment Dropout

Respondents who did not utilize any of the mental health services above were asked if there was a time during the previous twelve months when they felt the need to see a professional for mental health problems. They were classified either as *low perceived need* (if they did not think they needed help, or if they needed help for less than four weeks of last 12 months) or as having *perceived need* if they thought they needed help. The group that reported perceived need were then asked about *structural* and *attitudinal barriers* to seeking such help. 

Other respondents who accessed mental health services in the past twelve months were asked if the treatment had been terminated or if they had quit treatment before the provider had recommended termination. Those who had sought treatment but quit before treatment was completed were asked a complete a checklist of potential *structural or attitudinal* reasons for treatment dropout similar to that given to respondents who did not seek treatment. No further clarification was requested from those who reported improved health or no longer required help. Analyses were limited to those who dropped out of treatment prematurely and gave a reason for termination. If respondents reported more than one reason, then they were checked on each reason.

#### 2.2.5. Sociodemographic Predictor Variables

Sociodemographic variables included gender and age (15–65 years), as a continuous variable. Completed years of education classified as: *low* (0–6 years), *low-average* (7–9 years), *high-average* (10–15 years), and *high* (16+ years). These categories were based on levels of primary school, secondary school, high school, and three years into college, and college graduates. Family income status was calculated based on the household income divided by the number of people in that household and classified based on the median of the entire sample as follows: *low* (less than 50% of the median), *low-average* (values up to the median), *high-average* (between one and three times the median), and *high* (values more than three times the median). Marital status classified as *married, previously married* (separated, widowed or divorced), or *never married*.

### 2.3. Statistical Analysis

Part II data were weighted to adjust for the differential within-household probability of selection, differential nonresponse, and under-sampling of non-cases from Part I respondents into Part II. Descriptive statistics were calculated based on these weights. The distribution of sociodemographic variables (age, gender, education, income, and marital status), severity levels (severe, moderate, and mild), and disorders category (mood, anxiety, substance use, and impulse) were examined by service use status. The prevalence of reasons for not seeking treatment and for treatment dropout was calculated for both the total sample and those with perceived need. 

Multivariable logistic regression analyses were conducted to assess the association of sociodemographic variables and disorder severity with treatment status among those with 12-month DSM-IV/CIDI disorder, and low perceived need among those who did not use services. Models were adjusted for the sociodemographic variables, levels of severity, and disorders category. Due to the small samples size, only univariate analyses were possible for structural barriers among those who perceived the need. Logistic regression coefficients and their standard errors were exponentiated to produce odds-ratios (ORs) and their 95% confidence intervals (CIs). Wald chi-square statistic (χ^2^) was used for the multivariable significance tests and standard errors were calculated using the Taylor series method adjusting for clusters, stratification and weights. Statistical significance was evaluated using a two-sided design-based with 0.05-level tests. All analyses were performed using SAS^®^ software version 9.4 (SAS Institute Inc., Cary, NC, USA).

## 3. Results

### 3.1. Sociodemographics of Service Use

Of the 1981 Part II respondents, 711 (22.3%) met the criteria for at least one 12-month disorder of whom 597 (86.1%) reported no service use during that period (Table 1). The majority of the respondents with any diagnosed 12-month disorder were female (61.1%) and had a high-average level of education, (53.4%); 48.3% were never married, and 41.5% reported low household income. Higher proportions of low-income participants used professional services (46.8%) compared to those with high income (22.0%). The most commonly reported mental disorders were anxiety (55.0%) and mood disorders (51.1%). Among those who did not use services, approximately one third (32.5%) had mental health disorders classified as mild, with (33.8%) indicating severe illness. In contrast, the majority of respondents who reported using services (53.1%) had severe mental health conditions, with (27.6%) indicating mild illness. 

After adjustment for sociodemographic factors, severity level and disorder category, marital status was the only significant predictor for service use (Table 2). Relative to respondents who were never married, those who were previously married (OR 3.4, 95% CI 1.1–10.2) or currently married (OR 3.3, 95% CI 1.3–8.6) were significantly more likely to use mental health services. 

### 3.2. Barriers to Seeking Treatment

Around half (50.7%) of the 597 participants with 12-month DSM-IV/CIDI disorders who did not seek treatment, did not think they needed any help for their condition and were categorized as “low perceived need”. Of the 309 respondents (49.3%) who recognized a need to see a professional for mental health problems, i.e., had a “perceived need”, the vast majority (98.9%) reported attitudinal barriers to initiation (Table 3).

The by far most commonly cited attitudinal barriers among respondents with perceived need were wanting to handle the problem on their own (82.0%), followed by the belief that the problem was not severe (13.5%) and the perception that available services were ineffective (9.4%). In contrast, only 10.3% of people who perceived a need for treatment reported a primary structural barrier. Of those who perceived the need for service, 8.9% reported barriers related to availability and 7.3% barriers related to financial needs. Transportation and inconvenience were the least commonly reported structural barriers (5.8% and 6.4%, respectively).

Marital status was a significant predictor of low perceived need for treatment (Table 4). Relative to never-married respondents, those who were previously married were more likely to believe they needed help. Participants with low-average income were also more likely to perceive the need for treatment compared to participants in the high-income category. Among respondents with perceived need for mental health treatment, those who were previously married (OR 17.4, 95% CI 3.4–89.7) were more likely to report structural barriers relative to those who were never married (Table 5). Men participants were significantly less likely to report structural barriers relative to women. Moreover, structural barriers was significantly associated with severity (χ^2^ = 13.0). 

### 3.3. Reasons for Dropping out of Treatment

Of the 114 respondents with a 12-month DSM-IV/CIDI disorder who reported receiving mental health treatment during the past year, 26 dropped out of all treatment sectors and gave reasons for their dropout. Most reported at least one attitudinal barrier (85.2%) (Table 3), and indicated they did not need help anymore, i.e., were classified as “low perceived need” (56.1%); 20.0% reported structural barriers. All respondents who perceived a need for treatment (*n* = 10) reported at least one attitudinal barrier and 45.6% cited structural barriers. The most commonly reported attitudinal barrier was wanting to handle the problem on one’s own (83.0%). The multivariable analysis of treatment drop out could not be done due to the small sample size. 

## 4. Discussion

Findings of this study regarding barriers to treatment and reasons for early termination suggest that several factors may underlie the unmet service need documented in previous SNMHS publications [21]. These include personal perceptions of need for treatment, as well as attitudinal and structural barriers. Among all respondents with a mental health disorder, the most commonly reported barrier to seeking treatment was “low perceived need.” These results are consistent with findings from (WMH) surveys in 24 countries [12]. While reasons for low perceived need are unknown, possible explanations include: failure of respondents to recognize that they have a mental health disorder due to denial or lack of knowledge; receipt of other support or help from individuals (e.g., friend, family member) outside the healthcare settings assessed in the survey [30,31], and potential stigma associated with mental illness in certain segments of society [19].

On the other hand, among participants who perceived a need for treatment, the most commonly reported barriers to treatment were related to attitudinal factors (e.g., wanting to handle it on their own, not severe enough to seek help), followed by structural factors (e.g., service availability, financial constraints). This pattern of barriers indicates that the major determinants of unmet need for treatment in the KSA may be related more to individual beliefs and perceptions than the availability of mental health care access or delivery. In addition, a high proportion of people who use the services are designated as severe cases, suggesting that many people in the KSA may recognize the need for treatment only when their illness is severe.

In addition, we found that participants who were previously married were more likely to report using mental health services, to perceive a need for treatment, and to indicate structural barriers to treatment use than were those who were never married. Those who were currently married were more likely than never married participants to indicate the use of services, but not to report perceived treatment. This latter disparity might, in part, reflect greater social stability and access to social support among currently married adults or never married, potentially leading to a reduced perceived need for outside help. This possibility is supported by data showing that separated and divorced people report poorer physical and mental health compared to those who are married or single [32,33]. Although previous investigations have reported women to be more likely than men to recognize the need to seek treatment [34], we found no evidence for differences in perceived treatment need by gender in the current study. Attitudinal barriers were an obstacle for people who used the services but dropped out before the provider recommended termination. Unfortunately, predictors of attitudinal barriers could not be determined as the vast majority of respondents (98.9%) who perceived the need for treatment but did not receive any service, endorsed at least one such a barrier.

The health care system in the KSA is reasonably available as it provides free health care services. Moreover, the total number of mental health professionals working in the KSA is 19.3 per 100,000 population [35], and the rate of mental hospital beds is 17.1 per 100,000 population. These numbers are above the global median rate (9 per 100,000 population and 11.3 per 100,000 population, respectively) [36]. This could explain the low percentages of people reporting at least one structural barrier (5.1%) such as financial, availability, transportation, or inconvenience. Given the preponderance of explanations relating to perceived need, future work concerning the availability of mental health services in the KSA might benefit from a focus on the quality and marketing of services. Campaigns that promote free services are essential for people who believe services are not available to them. This also includes mental health literacy campaigns [37], which inform the public about critical reasons for seeking treatment. Special assistance programs for widows and divorcees have increased in KSA. These programs should not be limited to financial assistance but also to advocating mental health services when needed. Establishing a mental health care index that would measure and monitor the performance of services might encourage individuals to seek mental health services in the KSA. Moreover, optimizing the telehealth technology can reduce unmet mental health care need by providing access to mental health treatment, especially in remote areas [38].

This study has several limitations. First, the SNMHS is based on cross-sectional data which limits conclusions regarding causality. Data, including that on sensitive topics, are reliant primarily on self-report raising the possibility of recall and social desirability bias, and potentially leading to misclassification of certain variables. For example, since alcohol and drug use are illegal in the KSA, respondents may perceive that being truthful about their use of these substances involves admitting to a “crime’’, potentially rendering them less likely to report. Also, disorders were diagnosed retrospectively, which might underestimate their true prevalence. In addition, we restricted our analyses to respondents with a diagnosed 12-month disorder based on predefined criteria, which may have resulted in an underestimate of certain mental illnesses, potentially attenuating risk estimates and reducing generalizability. However, we believe that the use of this structured interview technique provides the most complete set of data on mental health available about the KSA population. Finally, some subgroup analyses were conducted with rather small sample sizes, reducing statistical power and potentially increasing margins of error; notably, analyses using relatively sparse data (e.g., structural barriers and dropout category) resulted in very wide confidence intervals. These limitations notwithstanding, the current study is the first of its kind in the KSA to examine the barriers to seeking mental health treatment and reasons for early treatment drop out using face-to-face interviews. Another strength of the study is the nationally-representative sample and used a valid and reliable tool of assessment DSM-IV/CIDI [39], facilitating comparisons between different countries.

## 5. Conclusions

Overall, this study found that attitudinal barriers rather than structural barriers (e.g., availability of services) were the primary obstacle for seeking treatment in the KSA. Previously married participants were more likely to report using mental health services, to perceive a need for treatment, and to indicate structural (rather than attitudinal) barriers to treatment use than were those who were never married. The results of the study suggest that mental health literacy is an important factor that might contribute to reducing unmet needs for the treatment.

## Figures and Tables

**Table 1:** Sociodemographic by 12-month service use among respondents with 12-month D Diagnostic and Statistical Manual of Mental Disorders (DSM-IV)/Composite International Diagnostic Interview (CIDI) disorders in the Saudi National Mental Health Survey.

	Service Use					
	Total (*n* = 711)		No (*n* = 597)		Yes (*n* = 114)	
**Age (years) Mean (SE)**	**29.1**	**(0.7)**	**28.3**	**(0.8)**	**33.4**	**(1.4)**
	**%**	**(SE)**	**%**	**(SE)**	**%**	**(SE)**
**Gender**						
Female	61.1	(3.1)	61.0	(3.3)	61.7	(8.7)
Male	38.9	(3.1)	39.0	(3.3)	38.3	(8.7)
**Education**						
Low	8.5	(1.2)	7.7	(1.4)	13.9	(5.3)
Low-average	16.9	(2.2)	16.3	(2.6)	20.1	(8.0)
High-average	53.4	(2.7)	54.8	(3.2)	45.0	(6.5)
High	21.1	(3.2)	21.1	(3.4)	21.0	(6.1)
**Income**						
Low	41.5	(2.9)	40.6	(3.1)	46.8	(9.9)
Low-average	9.1	(1.6)	8.5	(1.5)	13.0	(6.4)
High-average	19.2	(2.3)	19.4	(2.4)	18.2	(5.9)
High	30.1	(2.6)	31.4	(2.8)	22.0	(5.5)
**Marital status**						
Previously married	9.6	(2.4)	8.1	(2.1)	19.0	(7.4)
Currently married	42.1	(3.0)	39.7	(3.3)	56.8	(9.0)
Never married	48.3	(3.6)	52.2	(3.8)	24.1	(6.0)
**Severity**						
Severe	36.5	(2.6)	33.8	(2.7)	53.1	(9.4)
Moderate	31.7	(3.3)	33.7	(3.8)	19.3	(4.8)
Mild	31.8	(3.8)	32.5	(4.1)	27.6	(10.1)
**Mental disorders**						
Any mood	51.1	(2.7)	50.8	(2.9)	53.0	(7.3)
Any anxiety	55.0	(2.8)	52.8	(3.3)	69.0	(5.3)
Any substance use	8.4	(1.6)	5.5	(1.4)	26.3	(8.0)
Any impulse	24.3	(2.3)	25.2	(2.4)	19.0	(5.6)
**Total**	100.0	(0.0)	86.1	(2.2)	13.9	(2.2)

Abbreviations. SE, Standard Error: included to reflect uncertainty from weighted population sampling strategy.

**Table 2:** Sociodemographic correlates of mental health service use among respondents with twelve-month DSM-IV/CIDI disorders.

	Received Treatment (*n* = 114/597)		
	OR	(95% CI)	*p-*Value
**Age**	1	(1.0–1.0)	0.998
χ^2^_1_	0.0		
**Gender**			
Female	1	Ref	
Male	1.4	(0.6–3.0)	0.398
χ^2^_1_	0.7		
**Education**			
Low	1.0	(0.3–3.0)	0.900
Low-average	0.9	(0.3–3.0)	0.900
High-average	0.7	(0.3–1.5)	0.422
High	1	Ref	
χ^2^_3_	1.0		
**Income**			
Low	1.9	(0.6–5.5)	0.248
Low-average	1.6	(0.6–4.0)	0.329
High-average	1.3	(0.5–3.0)	0.564
High	1	Ref	
χ^2^_3_	1.6		
**Marital status**			
Previously married	3.4	(1.1–10.2)	0.030 *
Currently married	3.3	(1.3–8.6)	0.010 *
Never married	1	Ref	
χ^2^_2_	6.7		
**Severity**			
Severe	0.8	(0.2–3.1)	0.797
Moderate	0.4	(0.1–1.4)	0.180
Mild	1	Ref	
χ^2^_2_	3.4		
**Overall**			
χ^2^_16_	81.2 *		

Abbreviations. CIDI, Composite International Diagnostic Interview; OR, odds ratio; CI, confidence interval. * Significant at the 0.05 level, two-sided test. The models were estimated in the Part II sample. Analyses adjusted for all variables in the table plus 12-month mood disorders, 12-month anxiety disorders, 12-month substance disorders, and 12-month disruptive behavior disorder. Degrees of freedom are 1, 2, 3 and 16.

**Table 3:** Barriers to seeking treatment and reasons for dropping out of treatment in the past 12 months among respondents with 12-month DSM-IV/CIDI disorders (any severity).

	No Service Use			Drop Out	
	%	(SE)		%	(SE)
**I. Reasons for not seeking treatment (*n* = 597)**			**I. Reasons of dropping out (*n* = 26)**		
Low perceived need	50.7	(2.8)	Did not need help anymore	56.1	(12.2)
Structural barriers	5.1	(1.3)	Structural barriers	20.0	(11.3)
Attitudinal barriers	48.8	(2.8)	Attitudinal barriers	85.2	(5.5)
**II. Structural barriers among those with perceived need (*n* = 309)**			**II. Structural barriers among those with perceived need (*n* = 10)**		
Financial	7.3	(2.5)	Financial	43.1	(21.0)
Availability	8.9	(2.6)	Availability	14.8	(12.0)
Transportation	5.8	(2.3)	Transportation or Inconvenient	43.8	(20.9)
Inconvenient	6.4	(2.4)	Any	45.6	(20.7)
Any	10.3	(2.7)	**III. Attitudinal barriers among those with perceived need (*n* = 10)**		
**III. Attitudinal barriers among those with perceived need (*n* = 309)**			Wanted to handle on own	83.0	(10.6)
Wanted to handle on own	82.0	(3.1)	Perceived ineffectiveness	51.4	(19.2)
Perceived ineffectiveness	9.4	(2.5)	Stigma	29.2	(15.7)
Stigma	4.7	(2.2)	The problem got better	0.0	(0.0)
Thought would get better	8.7	(3.0)	Negative Experience with Treatment Provider	45.6	(20.7)
Problem was not severe	13.5	(3.3)	Any	100.0	(0.0)
Any	98.9	(0.6)			

Abbreviations. CIDI, Composite International Diagnostic Interview; SE, Standard Error: included to reflect uncertainty from weighted population sampling strategy.

**Table 4:** Sociodemographic correlates of not seeking treatment because of low perceived need among respondents with twelve-month DSM-IV/CIDI disorders.

	Low Perceived Need (*n* = 288/309)		
	OR	(95% CI)	*p-*Value
**Age**	1.0	(0.9–1.0)	0.270
χ^2^_1_	1.2		
**Gender**			
Female	1	Ref	
Male	0.9	(0.5–1.6)	0.662
χ^2^_1_	0.2		
**Education**			
Low	1.4	(0.4–3.1)	0.529
Low-average	1.2	(0.6–2.7)	0.591
High-average	1.6	(0.8–3.0)	0.153
High	1	Ref	
χ^2^_3_	2.3		
**Income**			
Low	1.0	(0.6–1.6)	0.864
Low-average	0.4	(0.1–1.0)	0.040 *
High-average	1.3	(0.6–2.7)	0.536
High	1	Ref	
χ^2^_3_	5.8		
**Marital status**			
Previously married	0.3	(0.1–0.9)	0.030 *
Currently married	1.4	(0.5–3.6)	0.466
Never married	1	Ref	
χ^2^_2_	14.7		
**Severity**			
Severe	0.6	(0.3–1.2)	0.144
Moderate	0.9	(0.5–1.7)	0.850
Mild	1	Ref	
χ^2^_2_	3.1		
**Overall**			
χ^2^_16_	33.6 *		

Abbreviations. CIDI, Composite International Diagnostic Interview; OR, odds ratio; CI, confidence interval. * Significant at the 0.05 level, two-sided test. The models were estimated in the Part II sample. Analyses adjusted for number of all variables in the table plus 12-month mood disorders, 12-month anxiety disorders, 12-month substance disorders and 12-month disruptive behavior disorders. Degrees of freedom are 1, 2, 3 and 16.

**Table 5:** Univariate analyses of sociodemographic correlates of not seeking treatment because of any structural barriers among respondents with twelve-month DSM-IV/CIDI disorders who perceived the need.

	Any Structural Barrier (*n* = 33/276)		
	OR	(95% CI)	*p-*Value
**Age**	1.0	(1.0–1.0)	0.728
χ^2^_1_	0.12		
**Gender**			
Female	1	Ref	
Male	0.2	(0.1–0.9)	0.028*
χ^2^_1_	4.8 *		
**Education**			
Low	2.3	(0.3–16.7)	0.115
Low-average	0.6	(0.1–7.1)	0.183
High-average	1.0	(0.3–3.2)	0.421
High	1	Ref	
χ^2^_3_	3.3		
**Income**			
Low	0.6	(0.2–2.0)	0.373
Low-average	0.7	(0.1–3.3)	0.641
High-average	0.3	(0.1–1.0)	0.057
High	1	Ref	
χ^2^_3_	3.7		
**Marital status**			
Previously married	17.4	(3.4–89.7)	<0.001 *
Currently married	3.2	(0.9–10.7)	0.064
Never married	1	Ref	
χ^2^_2_	11.7 *		
**Severity**			
Severe	1.9	(0.4–8.3)	0.387
Moderate	0.2	(0.0–1.3)	0.091
Mild	1	Ref	
χ^2^_2_	13.0 *		

Abbreviations. CIDI, Composite International Diagnostic Interview; OR, odds ratio; CI, confidence interval. * Significant at the 0.05 level, two-sided test. The models were estimated in the Part II sample. Degrees of freedom are 1, 2 and 3.
